# Cuproptosis depicts tumor microenvironment phenotypes and predicts precision immunotherapy and prognosis in bladder carcinoma

**DOI:** 10.3389/fimmu.2022.964393

**Published:** 2022-09-23

**Authors:** Huihuang Li, Xiongbing Zu, Jiao Hu, Zicheng Xiao, Zhiyong Cai, Ning Gao, Jinbo Chen

**Affiliations:** ^1^ Department of Urology, Xiangya Hospital, Central South University, Changsha, China; ^2^ National Clinical Research Center for Geriatric Disorders, Xiangya Hospital, Central South University, Changsha, China; ^3^ Department of Urology, Xiangya Boai Hospital, Changsha, China

**Keywords:** cuproptosis, bladder carcinoma, immunotherapy, prognosis, tumor microenvironment

## Abstract

**Background:**

Though immune checkpoint inhibitors (ICIs) exhibit durable efficacy in bladder carcinomas (BLCAs), there are still a large portion of patients insensitive to ICIs treatment.

**Methods:**

We systematically evaluated the cuproptosis patterns in BLCA patients based on 46 cuproptosis related genes and correlated these cuproptosis patterns with tumor microenvironment (TME) phenotypes and immunotherapy efficacies. Then, for individual patient’s evaluation, we constructed a cuproptosis risk score (CRS) for prognosis and a cuproptosis signature for precise TME phenotypes and immunotherapy efficacies predicting.

**Results:**

Two distinct cuproptosis patterns were generated. These two patterns were consistent with inflamed and noninflamed TME phenotypes and had potential role for predicting immunotherapy efficacies. We constructed a CRS for predicting individual patient’s prognosis with high accuracy in TCGA-BLCA. Importantly, this CRS could be well validated in external cohorts including GSE32894 and GSE13507. Then, we developed a cuproptosis signature and found it was significantly negative correlated with tumor-infiltrating lymphocytes (TILs) both in TCGA-BLCA and Xiangya cohorts. Moreover, we revealed that patients in the high cuproptosis signature group represented a noninflamed TME phenotype on the single cell level. As expected, patients in the high cuproptosis signature group showed less sensitive to immunotherapy. Finally, we found that the high and low cuproptosis signature groups were consistent with luminal and basal subtypes of BLCA respectively, which validated the role of signature in TME in terms of molecular subtypes.

**Conclusions:**

Cuproptosis patterns depict different TME phenotypes in BLCA. Our CRS and cuproptosis signature have potential role for predicting prognosis and immunotherapy efficacy, which might guide precise medicine.

## Introduction

Bladder carcinoma (BLCA) is among the most common carcinomas worldwide, with 549393 new patients diagnosed in 2018 (1). Muscle invasive bladder carcinomas (MIBCs) refer to the tumors that invade into the detrusor muscle. With higher rates to metastasize to lymph nodes or distant sites, patients with MIBCs possess extremely high disease specific mortality and need multimodal and invasive treatment (2). However, unlike large therapeutic improvements in other carcinomas, the therapeutic regimen for BLCA remained unchanged over the past 30 years (3). Although neoadjuvant chemotherapy (NAC) combined with radical cystectomy (RC) have become the golden treatment option, the prognoses of advanced BLCAs are still not satisfied with some patients insensitive to NAC or cisplatin intolerance (3). Fortunately, BLCA is a type of carcinoma with high tumor mutational burden (TMB) and immune checkpoint inhibitors (ICIs) including anti-PD-1 and anti-PD-L1 showed durable responses in a portion of patients (3–5). Thus, predicting which patients will be sensitive to ICIs treatments is the major task for improving the survival outcomes of advanced BLCAs.

To achieve this goal, understanding the effect mechanism behind ICIs is vital. Through targeting negative regulating receptors on effect immune cells (generally effect T cells), ICIs could reactivate and promote a durable antitumor response (6). Tumor microenvironment (TME), which is consisted of malignant and non-malignant cells (generally including stromal and immune cells), could be generally divided into two phenotypes based on the tumor-infiltrating lymphocytes (TILs) presence. Inflamed tumors (hot tumors) are those with high infiltration of TILs, while noninflamed tumors (cold tumors) are those with low infiltration (7). Theoretically, ICIs could only exhibit their efficacy on the presence of T cells and would show higher response rates in the inflamed tumors (8). Actually, with scarce TILs infiltration, noninflamed tumors like prostate cancer and glioblastoma are resistant to the treatment of ICIs (9–11). While for the tumors with high TILs infiltration, patients show significantly higher response to ICIs treatment (12, 13). As a result, distinguishing noninflamed tumors from inflamed could not only have the potential for predicting ICIs efficacy, but also for turning “cold” into “hot” for higher ICIs efficacy (8). For BLCA, there are molecular subtypes exhibiting different response rates to ICIs, like basal subtypes showing higher response rates than luminal subtypes (14, 15).

Copper (Cu) is a mineral nutrient and its imbalance is related with pathologies like Wilson disease and proliferative, angiogenesis and metastasis of carcinoma (16). Recently, Tsvetkov et al. made a breakthrough that excess of intracellular copper concentrations could induce a unique type of cancer cell death which is distinct from other programmed cell deaths like apoptosis, pyroptosis, necroptosis and ferroptosis, namely cuproptosis (17). Therefore, genes involved in copper homeostasis and cuproptosis could play key roles in the cancer developments and cancer immune processes (18). Copper transporter 1 (CTR-1), as the most important copper influx transporter, was found to be significant related with PD-L1 expression and play vital role in the cancer immune evasion (19). In addition, MT1 was reported to induce an immunosuppressive phenotype by inducing tolerogenic dendritic cells (DCs) (20). Cytochrome c oxidase 17 (COX17) played vital role in the cancer development and its knockdown could inhibit the invasion and metastasis of triple negative breast cancer (TNBC) (21). However, comprehensive analysis of these cuproptosis related genes is still lacking. In this study, for the first time, we comprehensively correlated 46 cuproptosis related genes with TME phenotypes, precision immunotherapy efficacy and prognosis in BLCA.

## Materials and methods

### Data sources

The fragments per kilobase per million mapped fragments (FPKM), count value and clinical data of TCGA-BLCA was downloaded from UCSC Xena (https://xenabrowser.net/). The FPKM value was transformed into transcripts per kilobase million (TPM) value and duplicated patients or patients without matched RNA-seq data and survival data were excluded. Finally, 411 tumor samples and 19 normal samples in TCGA-BLCA were included for further analysis. Xiangya cohort (GSE188715) was developed as our previous study reported (22). Another two databases (GSE32894 and GSE13507) were downloaded from GEO database (https://www.ncbi.nlm.nih.gov/geo/) using “getGEO” function in the “GEOquery” R package. Immunotherapy datasets including GSE173839, GSE135222 and GSE100797 were downloaded from GEO database using the same method, while PMID26359337 was downloaded from the corresponding supplementary material of the study (23).

### Unsupervised clustering

46 cuproptosis related genes were collected from the studies of Tsvetkov et al. (17) and Ge et al. (18). We conducted consensus clustering and repeated 1000 times using “ConsensuClusterPlus” R package (maxK=4, reps=1000, pItem=0.8, distance=“euclidean”, clusterAlg=“km”). Cuproptosis related genes were summarized in **Table S1**.

### Pathway enrichment analysis

Four immune-related signatures and 21 signatures related to efficacy of immune checkpoint blockade (ICB) therapy were collected from previous studies (15, 24). Other therapeutic signatures and Drugbank database were also collected as reported in our previous study (25). Additionally, signatures related to molecular subtypes of BLCA were collected from Kamoun’s study (14). Then we calculated the sample-level enrichment scores of these signatures using single sample gene set enrichment analysis (ssGSEA) implemented in “GSVA” R package.

Differential gene expression analysis was conducted using empirical Bayesian algorithm (“limma” R package) and the criteria for differentially expressed genes (DEGs) was set as absolute log2 fold change (FC) greater than 2.5 and adjust *p* value less than 0.05. Hallmark, gene ontology (GO) and Kyoto Encyclopedia of Genes and Genomes (KEGG) gene sets were downloaded from MSigDB (26) (http://www.gsea-msigdb.org/gsea/index.jsp) and then GSEA analysis was performed using “GSVA” R package.

### Tumor immune microenvironment depiction

Anti-cancer immunity cycle describes a seven-step-based process for anti-tumor immune cells activation and the levels of each step were downloaded from the tracking tumor immunophenotype (TIP) (http://biocc.hrbmu.edu.cn/TIP/) as our previous study described (22). Also, the relative abundances of 28 immune cells in TCGA-BLCA and Xiangya cohort were calculated using ssGSEA algorithm based on the gene sets reported in Charoentong’s study (27).

### Development and validation of a cuproptosis risk score

Univariable Cox regression model was applied to filter the prognostic genes based on DEGs and the filtered prognostic genes were further narrowed down using the least absolute shrinkage and selector operation (LASSO) and ten-fold cross validation. Then a CRS was developed by Cox proportional hazard regression algorithm using the “glmnet” R package:


CRS = Σβі*RNAi

External databases including GSE32894 and GSE13507 were used to validate this risk score.

### Single cell RNA sequencing

A scRNA-seq dataset containing seven BLCA samples was downloaded from the supplemental material of GSE135337 (28). The raw count matrixes were used to create Seurat object using “Seurat” R package and the inclusion criteria for high quality cells were set as: numbers of unique molecular identifier (UMI) more than 1000, genes more than 250, log10GenesPerUMI more than 0.80 and mitochondrial percent less than 20%. Then the raw data count was normalized, identified variable genes and scaled using SCTransform function. Seven samples were integrated based on Anchors generated by the top 3000 variables (FindIntegrationAnchors function). After integration, RunPCA function was used to conduct principal component analysis (PCA) and the top 40 PCs were then used to perform uniform manifold approximation and projection (UMAP) reduction. FindClusters function was conducted to identify the main cell clusters with a resolution value of 0.4 and the cell clusters were annotated based on the gene markers reported as previous study (29). AddModuleScore function was used to generate a cuproptosis signature on the single cell level.

### Assessment of molecular subtypes of BLCA

“ConsensusMIBC” and “BLCAsubtyping” R packages were used to divided BLCA patients into different molecular subtypes. In addition, we regrouped these subtypes as dichotomous outcomes, namely “basal” and “luminal” subtypes, according to the consensus subtype (14). Detailed description could be found in our previous studies (22, 25).

### Statistical analysis

If continuous variables fitted normally distributed criterion, unpaired t-test was applied to compare the differences, otherwise Wilcoxon rank-sum test was applied. χ2 or Fisher’s exact test was applied to compare categorical values. The survival curves and the survival differences between two groups were generated using Kaplan-Meier method and log-rank tests respectively (“survminer” R package). Pearson correlation coefficients was applied to conduct correlation analyses. The predictive accuracy was judged using time-dependent receiver operating characteristic (ROC) analysis (timeROC function in the “tROC” R package). P values for DEG and GSEA analyses were adjusted using false discovery rate (FDR) method. Two-tailed *p* value less than 0.05 was set as the significant different criteria and all the analyses were finished using R 4.1.3.

## Results

### Cuproptosis regulated patterns in BLCA

**Figure 1:**
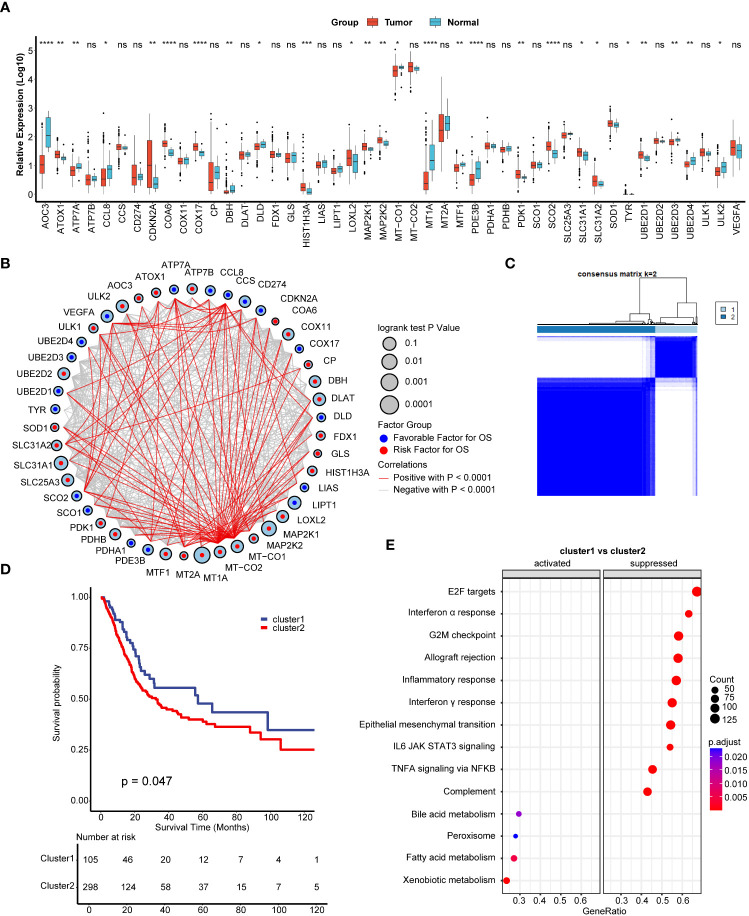
Development of cuproptosis regulated patterns in bladder carcinoma (BLCA). **(A)** The expression of 46 cuproptosis related genes between normal and BLCA tissues. Tumor, red; Normal, azure. **(B)** The comprehensive interactions between cuproptosis related genes. The size of circles represented the different effects of genes on the prognosis. Blue dots in the circles represented favorable factors for overall survival (OS), while the red dots represented risk factors. **(C)** Two clusters were generated by unsupervised clustering based on 36 cuproptosis related genes. **(D)** Kaplan-Meier plots between two cuproptosis regulated patterns. Blue line represented cuproptosis cluster1, while red line represented cuproptosis cluster2. **(E)** Gene set enrichment analysis (GSEA) of hallmark pathways between cuproptosis cluster1 and cluster2. *p < 0.05; **p < 0.01; ***p < 0.001, ****p < 0.0001. ns, not significant.

We found a majority of cuproptosis related genes were dysregulated between BLCA carcinoma and paired normal tissues (**Figure 1A**). Among these dysregulated genes, for example, COX17 plays vital role in the development of TNBC (21), while SLC31A1 is a key target gene for chemoresistance in ovarian cancer (30), indicating cuproptosis related genes might also play vital roles in BLCA. We further depicted that cuproptosis related genes had a closed relationship among each other and most of these genes had prognostic value in BLCA (**Figure 1B**). Inspired by these results, we wondered if cuproptosis related genes had a comprehensive regulated pattern in BLCA and conducted unsupervised clustering. We found that TCGA-BLCA patients could be well divided into two clusters, named cuproptosis cluster1 and cluster2 (**Figure 1C**, **Figures S1A–D**). Importantly, patients in cuproptosis cluster1 exhibited significantly favorable survival outcomes than patients in cluster2 (**Figure 1D**). GSEA results of hallmark pathways revealed that a majority of immune related pathways (including inflammatory, interferon α and γ response) were suppressed in cuproptosis cluster1 (**Figure 1E**, **Table S2**), indicating that there might be a different immune status between these two clusters.

### Different immune characteristics between cuproptosis regulated patterns

**Figure 2:**
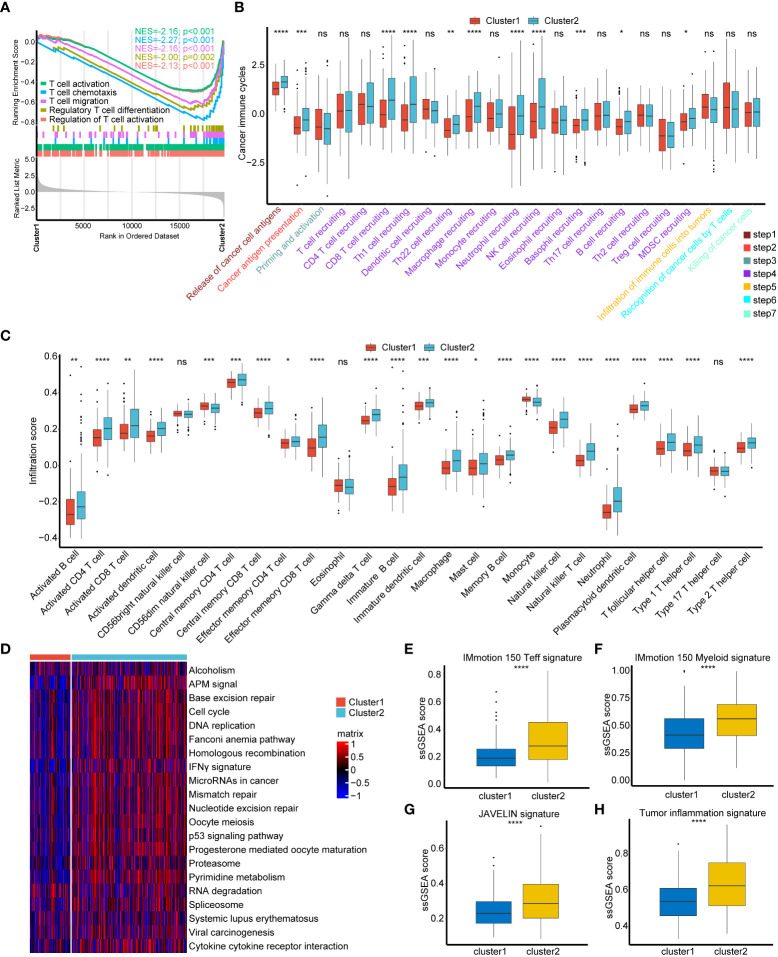
Consistency between cuproptosis patterns, tumor microenvironment (TME) phenotypes and immunotherapy efficacy. **(A)** T cell regulated pathways in gene ontology (GO) pathways using GSEA analysis. **(B)** Different activation status of cancer immune cycles between two cuproptosis regulated patterns. Tumor, red; Normal, azure. **(C)** Different infiltration status of immune cells into TME between two cuproptosis regulated patterns. Tumor, red; Normal, azure. **(D)** Heatmap of 21 pathways related to immunotherapy efficacy between two cuproptosis regulated patterns. The darker the red, the more pronounced activation of pathways. The darker the blue, the more pronounced inhibition of pathways. **(E–H)** Box plots of IMmotion 150 T-effector response **(E)**, IMmotion 150 Myeloid inflammatory **(F)**, JAVELIN **(G)** and tumor inflammation **(H)** gene expression signatures respectively between between two cuproptosis regulated patterns. *p < 0.05; **p < 0.01; ***p < 0.001, ****p < 0.0001. ns, not significant.

To our surprise, all positive regulation of T cell activation pathways were significantly suppressed in cuproptosis cluster1 (**Figure 2A**, **Table S2**). We were wondering if cluster1 represented a non-inflamed TME phenotype of BLCA and then compared cancer immune cycles between these two clusters. As showed in **Figure 2B**, a majority of cancer immune cycles were significantly lower in cuproptosis cluster1 than cluster2, indicating that patients in cluster1 might inhibit cancer immune activation and immune cells infiltration into TME. ssGSEA results further revealed that most TILs like activated CD4^+^ T cells, CD8^+^ T cells, dendritic cells (DCs) and natural killer (NK) cells were significantly lower in cuproptosis cluster1 (**Figure 2C**). These results supported that cuproptosis cluster1 represented a non-inflamed TME phenotype and would be insensitive to ICIs treatment, while cluster2 represented an inflamed phenotype and could be sensitive to ICIs. Mariathasan et al. summarized 21 pathways possessing immunotherapy efficacy predicting value (15) and we found all these pathways were inhibited in cuproptosis cluster1 (**Figure 2D**). In addition, another four immune related pathways including IMmotion150 T-effector (Teff) signature, IMmotion150 Myeloid signature, JAVELIN signature, and Tumor inflammation signature (TIS) were all significantly suppressed in cuproptosis cluster1 (**Figures 2E–H**). These results indicated that cuproptosis cluster1 might be insensitive to ICIs treatment. However, unsupervised clustering was conducted based on a cohort of patients and could not evaluate the regulation pattern of a single patient. So, we were aimed to screen out novel genes to predict individual patient’s prognosis, TILs infiltration, and immunotherapy efficacy.

### Construction and validation of cuproptosis risk score and its clinical significance

**Figure 3:**
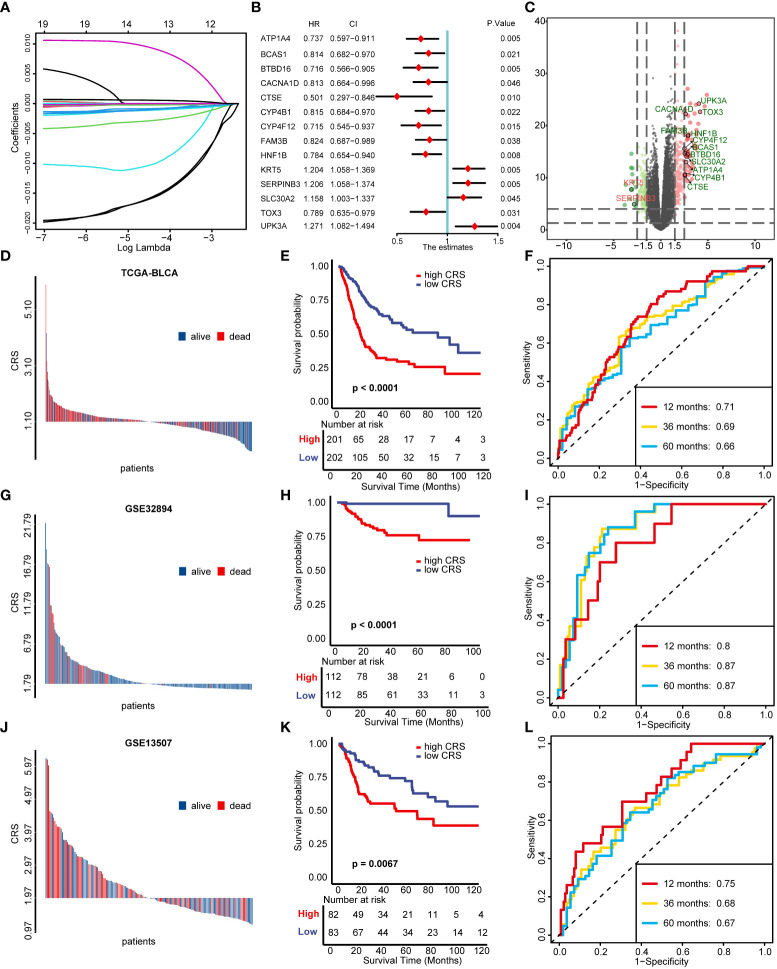
Construction and validation of cuproptosis risk score (CRS). **(A)** LASSO regression of the 19 genes possessing prognostic value. **(B)** Univariable cox regression analysis results of 14 genes selected for developing CRS. **(C)** The volcano plot of the 14 genes selected for developing CRS between two cuproptosis regulated patterns. **(D-F)** Distribution of patients’ survival status **(D)**, Kaplan–Meier survival plot **(E)** and time-dependent ROC curves of CRS **(F)** in TCGA-BLCA cohort. **(G-I)** Distribution of patients’ survival status **(G)**, Kaplan–Meier survival plot **(H)** and time-dependent ROC curves of CRS **(I)** in GSE32894 validation cohort. **(J-L)** Distribution of patients’ survival status **(J)**, Kaplan–Meier survival plot **(K)** and time-dependent ROC curves of CRS **(L)** in GSE13507 validation cohort.

We screened out 69 DEGs between cuproptosis cluster1 and cluster2 (**Table S3**) and further performed univariable cox regression model to select 20 genes possessing prognostic value (**Table S4**). Among these 20 genes, ACSM6 was ruled out because of none expression in all other GEO databases and the genes left were further narrowed down using LASSO and ten-fold cross validation (**Figure 3A**, **Figure S2A**). 14 genes were finally screened out to construct CRS and the univariable prognostic values of these genes were shown in **Figure 3B**. All these genes except KRT5 and SERPINB3 were significantly upregulated in cuproptosis cluster1 compared with cluster2 (**Figure 3C**). Then, a CRS was constructed using cox proportional hazard regression algorithm based on these genes: CRS = (-0.15)*ATP1A4 + (-0.04)*BCAS1 + (-0.13)*BTBD16 + (-0.06)*CACNA1D + (-0.36)*CTSE + (-0.05)*CYP4B1 + (-0.14)*CYP4F12 + (-0.02)*FAM3B + (-0.22)*HNF1B + 0.05*KRT5 + 0.13*SERPINB3 + 0.29*SLC30A2 + (-0.04)*TOX3 + 0.34*UPK3A. Cuproptosis cluster2 showed significantly higher CRS than cluster1 (**Figure S2B**, p< 0.001). Patients with higher CRS exhibited significantly poorer survival outcomes in TCGA-BLCA training cohort (**Figures 3D, E**, p< 0.0001) and the predictive accuracies were satisfied, with area under curve (AUC) around 0.70 (**Figure 3F**). Importantly, the CRS could be well validated in external cohorts including GSE32894 and GSE13507. In GSE32894, patients with higher CRS showed poorer survival outcomes (**Figures 3G, H**, p< 0.0001) with extremely high predictive accuracy (**Figure 3I**, AUCs at 12, 36, 60 months were 0.80, 0.87 and 0.87 respectively). The same in GSE13507, patients with higher CRS showed poorer survival outcomes (**Figures 3J, K**, p = 0.0067) with high predictive accuracy (**Figure 3L**, AUCs at 12, 36, 60 months were 0.75, 0.68 and 0.67 respectively). These results revealed that our CRS could be a novel tool for predicting individual BLCA patient’s prognosis.

We then performed univariable Cox regression analysis and found that older age, higher tumor stage and CRS were risk factors for OS (**Figure S2C**). Also, all these factors remained independent risk factors in multivariable Cox regression analysis (**Figure S2D**). So, we incorporated age, tumor stage and CRS to develop a nomogram for clinical application (**Figure S2E**). As showed in **Figure S2F**, the OS outcomes predicted by nomogram were generally consistent with the actual outcomes. Moreover, our nomogram exhibited the highest predictive accuracy, with AUC values of 0.73, 0.70 and 0.68 in 1, 3 and 5 years respectively (**Figures S2G–I**).

### Cuproptosis signature for individual patient’s immune cell infiltration evaluation

For individual patient’s immune cell infiltration evaluation, we first performed a systematically correlation analysis of the 14 novel genes and immune cells. To our surprise, KRT5 and SERPINB3, which were significantly downregulated in cuproptosis cluster1 (**Figure 3C**), were correlated with a specific inflamed TME phenotype both in TCGA-BLCA and Xiangya cohort (**Figures S3A, B**). The remaining other 12 novel genes, which were significantly upregulated in cuproptosis cluster1 (**Figure 3C**), were all correlated with a specific noninflamed TME phenotype in both TCGA-BLCA and Xiangya cohort (**Figures S3A, B**). These results further indicated that cuproptosis cluster1 and cluster2 represented noninflamed and inflamed TME phenotypes respectively. So, we constructed a cuproptosis signature for predicting individual patient’s TILs infiltration based on these 14 genes using ssGSEA algorithm.

**Figure 4:**
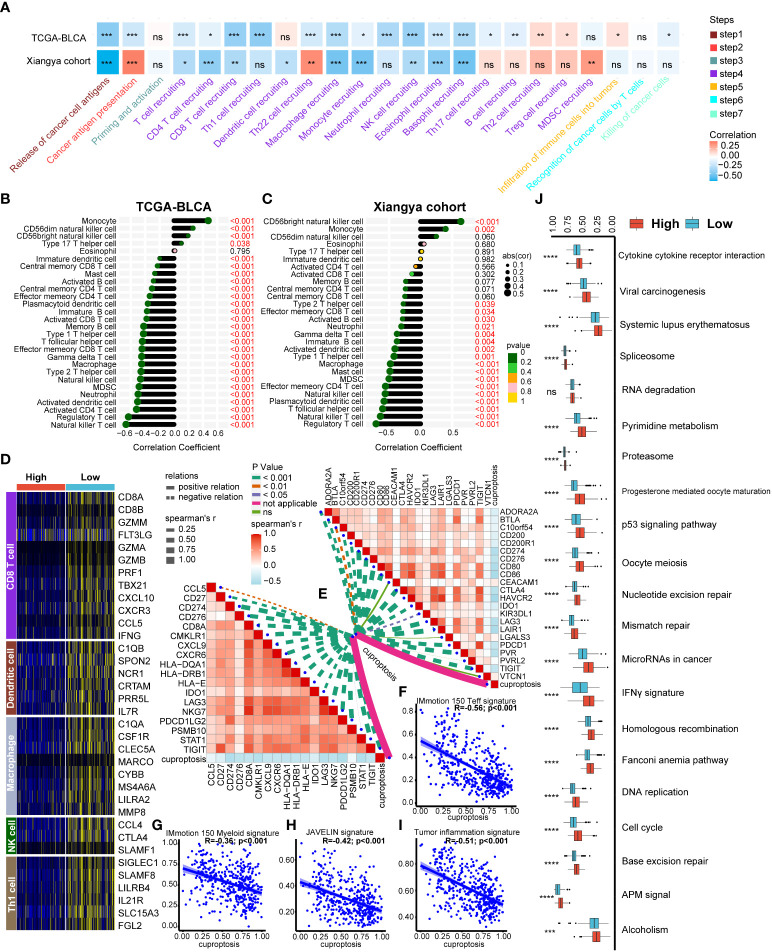
Developing a cuproptosis signature for individual patient’s tumor microenvironment (TME) phenotypes evaluation. **(A)** Correlation heatmap between cuproptosis signature and cancer immunity cycles in the TCGA-BLCA and Xiangya cohorts respectively. **(B-C)** Correlation between cuproptosis signature and immune cells infiltration in TCGA-BLCA **(B)** and Xiangya cohorts **(C)** respectively. **(D)** Heatmap of effect genes of CD8^+^ T cell, dendritic cell (DC), macrophage, natural killer (NK) cell and type 1 T helper (Th1) cell between high and low cuproptosis signature groups. **(E)** Correlation between cuproptosis signature, immune checkpoint (ICI) genes (topper right) and tumor inflammation signature (TIS) genes (lower left) respectively. **(F-I)** Correlation between cuproptosis signature, IMmotion 150 T-effector response **(F)**, IMmotion 150 Myeloid inflammatory **(G)**, JAVELIN **(H)** and tumor inflammation **(I)** gene expression signatures respectively. **(J)** Enrichment of each immunotherapy related pathways between high and low cuproptosis signature groups. *p < 0.05; **p < 0.01; ***p < 0.001, ****p < 0.0001. ns, not significant.

As we expected, a majority of cancer immune cycles including release of cancer antigens, T cell recruiting and NK cell recruiting were all significantly negative correlated with cuproptosis signature both in TCGA-BLCA and Xiangya cohort (**Figure 4A**, **Table S5**). In addition, TILs infiltration calculated using ssGSEA algorithm were generally negative correlated with our signature both in TCGA-BLCA (**Figure 4B**, **Table S5**) and Xiangya cohort (**Figure 4C**, **Table S5**) including activated CD4^+^ T cell, CD8^+^ T cell, DCs and NK cells. Moreover, the negative relationship between cuproptosis signature and immune cells infiltration could also be well validated in GSE32894 and GSE13507 (**Figures S4A, B**). As reported in our previous studies (22, 25), effector genes of CD8^+^ T cells, DCs, macrophage, NK cells, and type 1 helper (Th1) cells were summarized and all the genes were upregulated in the low cuproptosis signature group (**Figure 4D**). What’s more, cuproptosis signature was negative correlated with 22 ICIs genes (**Figure 4E**, right upper; **Table S6**) and 18 TIS genes (**Figure 4E**, left bottom; **Table S6**). For immunotherapy efficacy predicting, our signature was significantly negative correlated with IMmotion150 Teff signature (**Figure 4F**, R=-0.56, p<0.001), IMmotion150 Myeloid signature (**Figure 4G**, R=-0.36, p<0.001), JAVELIN signature (**Figure 4H**, R=-0.42, p<0.001), and TIS (**Figure 4I**, R=-0.51, p<0.001). Moreover, all 21 pathways related to efficacy of ICIs treatment were significantly upregulated in the low cuproptosis signature group (**Figure 4J**). Besides immunotherapy predicting, our signature could be also used to predict other therapeutic opportunities. As showed in **Figure S5A**, patients with lower cuproptosis signature were more likely to response to chemotherapy, immunotherapy and ERBB therapy while patients with higher signature could benefit more from antiangiogenic therapy according to Drugbank database. Immunosuppressive oncogenic pathways including PPARG network, WNT β catenin network, FGFR3 coexpressed genes and et al. were all upregulated in patients with higher cuproptosis signature (**Figure S5B**). However, EGFR network and radiotherapy predicted pathways were upregulated in patients with lower cuproptosis signature (**Figure S5B**), indicating these patients could be more sensitive to EGFR or radiotherapy treatment. In sum, we built a cuproptosis signature which could predict individual patient’s immune cell infiltration and potential therapeutic opportunities.

### The role of cuproptosis signature on the single cell level

**Figure 5:**
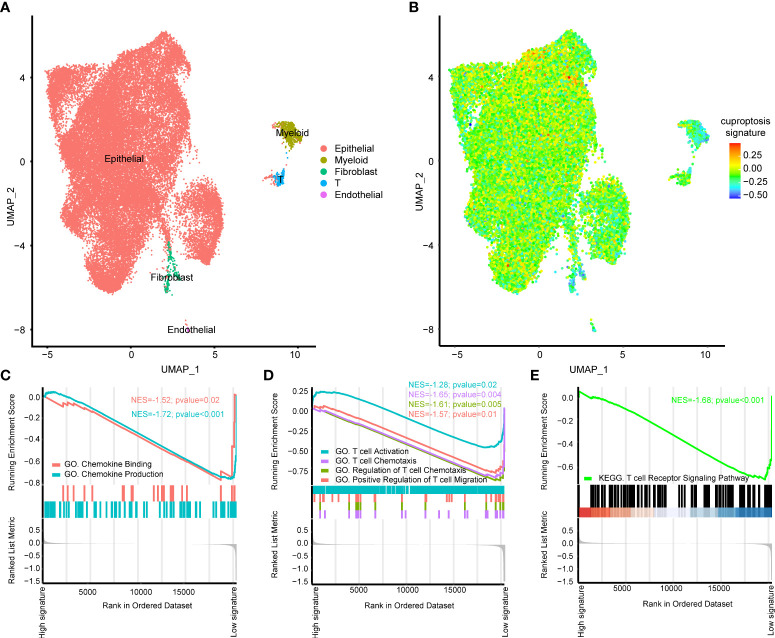
The role of cuproptosis signature on the single cell level. **(A)** UMAP plots of GSE135337 and each cluster was visualized and marked by different cell types. **(B)** Distribution of cuproptosis signature on the single cell level. **(C)** Gene ontology (GO) enrichment of chemokine **(C)** and T cell activation **(D)** related signatures identified by gene set enrichment analysis (GSEA) on the single cell level. **(E)** Kyoto Encyclopedia of Genes and Genomes (KEGG) enrichment of T cell activation related signatures identified by GSEA on the single cell level.

All the above analysis were based on bulk RNA-seq, then we aimed to figure out if our cuproptosis signature had immune predicting value on the single cell level using scRNA-seq. As shown in **Figure 5A**, seven BLCA samples from public dataset could be well annotated into epithelial (EPCAM+), myeloid (LYZ+), fibroblast (COL1A1+), T (CD3D+) and endothelial cells (CD31+) (28, 29). Obviously, cuproptosis signature was specifically high in epithelial cells (**Figure 5B**) and we further chose epithelial cells for the subsequent analysis. Chemokine related pathways including chemokine binding and chemokine production were significantly downregulated in the high cuproptosis signature group (**Figure 5C**, **Table S7**). Furthermore, T cell activation related pathways were all downregulated in the high cuproptosis signature group both in GO (**Figure 5D**, **Table S7**) and KEGG analysis (**Figure 5E**, **Table S7**).

### Cuproptosis signature for predicting immunotherapy efficacy and molecular subtypes of BLCA

**Figure 6:**
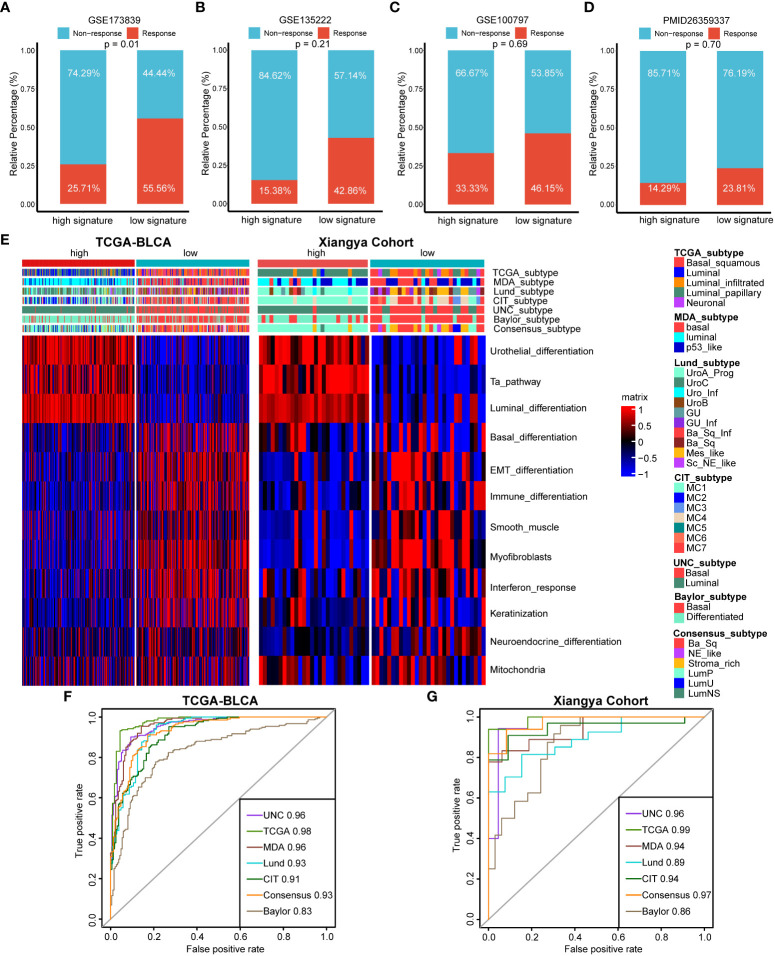
The roles of cuproptosis signature for predicting immunotherapy efficacy and molecular subtypes of BLCA. **(A–D)** Cuproptosis for predicting immunotherapy efficacy in GSE173839 **(A)**, GSE135222 **(B)**, GSE100797 **(C)** and PMID26359337 **(D)** datasets. **(E)** Distribution of molecular subtypes of BLCA and bladder cancer related pathways between high and low cuproptosis signature groups in TCGA-BLCA and Xiangya cohort respectively. **(F, G)** ROC curves of cuproptosis signature for predicting molecular subtypes of BLCA in TCGA-BLCA and Xiangya cohort respectively.

For immunotherapy efficacy predicting, we divided patients into response group including complete response (CR) and partial response (PR) patients and non-response group including stable disease (SD) and progressive disease (PD) patients. We found that there were significantly more response patients in the low cuproptosis signature group in GSE173839 (**Figure 6A**, p=0.01). Although without statistic difference, patients in the low cuproptosis signature group showed an obvious trend of higher response rates to immunotherapy in GSE135222, GSE100797 and PMID26359337 cohorts (**Figures 6B–D**). So, our cuproptosis signature could not only predict TILs infiltration, but also directly predict immunotherapy efficacy.

For BLCA, there are molecular subtypes exhibiting different response rates to immunotherapy treatment, like basal subtypes exhibiting poorer survival outcomes and higher immunotherapy response rates than luminal subtypes. In agreement with previous results, high cuproptosis signature group represented a luminal subtype enriched for urothelial differentiation, Ta and luminal differentiation pathways, while low cuproptosis signature group represented a basal subtype enriched for basal related pathways like basal differentiation and epithelial-mesenchymal transition (EMT) differentiation pathways both in TCGA-BLCA and Xiangya Cohort (**Figure 6E**). Importantly, the predictive accuracies of cuproptosis signature for basal and luminal subtypes predicting were both extremely high in TCGA-BLCA and Xiangya Cohort (**Figures 6F, G**), with all AUC values around 0.90. These results revealed that our cuproptosis signature possessed TILs infiltration and immunotherapy efficacy predicting values on the aspect of molecular subtypes of BLCA.

## Discussion

Recently, a unique type of cancer cell death distinct from other programmed cell deaths like apoptosis, pyroptosis, necroptosis and ferroptosis, namely cuproptosis, was reported by the study of Tsvetkov et al. and caught many researchers’ attention (17). In fact, as a mineral nutrient, copper was wildly reported to be related with pathologies of carcinoma (16). Eva et al. summarized the genes involved in copper homeostasis and a majority of these genes were reported to play vital role in the cancer development and cancer immune evasion (18). Among these genes, for example, lysyl oxidase (LOX) family contributes to the angiogenesis, metastasis and formation of extracellular matrix (ECM) in carcinomas (31). Specifically, LOX2 was found to increase ECM and prevent CD8^+^ T cells infiltration into TME, thus causing patients’ resistance to anti-PD-L1 (32). Downregulation of amine oxidase copper containing 3 (AOC3) was reported to decrease immune cell recruitment and promote lung cancer progression (33). Importantly, as the most important copper influx transporter, copper transporter 1 (CTR-1) was found to be significantly related with PD-L1 expression and play vital role in the cancer immune evasion (19). However, all these studies focused on only one or a small set of novel genes, and the comprehensive relationships among cuproptosis related genes, cancer immune and immunotherapy are lacking.

In our previous study, we generated m6A modification clusters and correlated them with TME and immunotherapy efficacy in renal carcinoma based on 24 m6A regulator genes (34). There are many other studies focusing on the relationships among TME and lists of genes, indicating the complexity and coregulated features of TME. Wan et al. constructed two ferroptosis clusters and systematically analyzed their relationships with prognosis and immunotherapy efficacy in glioma (35). Cao et al. divided BLCA patients into five hypoxia response patterns and individual hypoxia response pattern was also generated (36). Their findings could reveal the immune escape mechanism and promote the precise immunotherapy application for BLCA. In lung adenocarcinoma (LUAD), Liu et al. evaluated the m5C modification patterns and found three clusters with vital role in the TME regulation (37). As far as we known, this is the first study systematically correlating cuproptosis related genes with TME, prognosis and immunotherapy efficacy in BLCA. We identified two cuproptosis clusters and found there were different survival outcomes behind these two clusters. Moreover, different cuproptosis clusters represented different TME phenotypes and immunotherapy response rates. For individual BLCA patient, we also constructed CRS and cuproptosis signature for prognosis and cancer immune predicting respectively.

BLCA, as the most common carcinomas worldwide, causes huge threaten to human being’s health and economy: For non-muscle invasive bladder cancer (NMIBC), its high rate of recurrence and progression committing long-term invasive surveillance. While the high metastasis potential of MIBC makes it high disease specific mortality (2). What’s more, about 15% to 20% of NMIBC will develop into MIBC diseases, which possess even worse survival outcomes and poorer response to NAC therapy compared with primary MIBCs (3, 38, 39). Because of BLCA’s high TMB, ICIs treatment showed durable efficacy in a portion of patients and the US Food and Drug Administration (FDA) has already approved five ICIs for BLCA treatment (3). Rosenberg et al. reported the first clinical trial of atezolizumab in advanced BLCA and showed an overall objective response rate (ORR) for 15%. In addition, they also correlated TCGA molecular subtypes with immunotherapy efficacy (4). Sharma et al. investigated the role of nivolumab monotherapy in advanced BLCA and found nivolumab possessing 24.4% overall ORR with acceptable treatment-related adverse events (40). Bellmunt et al. found that pembrolizumab could significantly improve the OS outcomes of advanced BLCA (41). All these clinical trials promoted the approvements of ICIs for BLCA and revealed the significant roles of ICIs. However, not all patients in these trials responded to ICIs treatment, indicating urgent needs for discovering biomarkers predicting ICIs’ efficacies.

Recently, numerous studies found that TME contributed to the cancer biology and immunology by influencing host’s immune system (8, 42–44). Patients with immunosuppression microenvironments and less TILs showed poorer response rates to ICIs treatment and poorer OS (4, 45). Thus, distinguishing noninflamed tumors from inflamed could not only have the potential for predicting ICIs efficacy, but also for turning “cold” into “hot” for higher ICIs efficacy (8). There are numerous studies correlating pyroptosis, ferroptosis and tertiary lymphoid structure signatures with TME and immunotherapy efficacy in BLCA (46–48). In this study, we generated a cuproptosis signature for precise TME phenotypes predicting for the first time. Importantly, this result could be well validated in our own cohort, which made our result more believable. What’s more, our cuproptosis signature could directly predict immunotherapy efficacy, which could be vital for precise ICIs treatment in BLCA.

Limitations for our study: First, all of our results were generated from retrospective data and need further validations using prospective studies. Second, although we validated the role of cuproptosis signature in TME and immunotherapy, the detailed mechanisms should be further investigated *in vitro* and *in vivo*.

## Conclusion

Cuproptosis patterns depict different TME phenotypes in BLCA. Our CRS and cuproptosis signature have potential role for predicting prognosis and immunotherapy efficacy, which might guide precise medicine.

## Data availability statement

Publicly available datasets were analyzed in this study. This data can be found here: https://portal.gdc.cancer.gov/; https://www.ncbi.nlm.nih.gov/geo/.

## Ethics statement

This study was reviewed and approved by Medical Ethics Committee of Xiangya Hospital Central South University, GCP number 202104145. Written informed consent was obtained from all participants for their participation in this study.

## Author contributions

HL, XZ, JH, ZX performed analyses and drafted the manuscript. HL, XZ, JH and ZX searched and downloaded the original datasets from TCGA and GEO. HL, JH and ZC contributed to statistical analyses. HL, NG and JC edited the pictures. NG and JC conceived and supervised the study. All authors contributed to writing the manuscript. All authors contributed to the article and approved the submitted version.

## Funding

This work was supported by the National Natural Science Foundation of China (81873626, 81902592, 82070785), Hunan Natural Science Foundation (2020JJ5884) and Hunan Province Young Talents Program (2021RC3027).

## Conflict of interest

The authors declare that the research was conducted in the absence of any commercial or financial relationships that could be construed as a potential conflict of interest.

## Publisher’s note

All claims expressed in this article are solely those of the authors and do not necessarily represent those of their affiliated organizations, or those of the publisher, the editors and the reviewers. Any product that may be evaluated in this article, or claim that may be made by its manufacturer, is not guaranteed or endorsed by the publisher.

## Supplementary material

Supplementary Figure 1 Unsupervised clustering of 46 cuproptosis related genes in the TCGA-BLCA cohort.

Supplementary Figure 2 Construction of a nomogram by integrating cuproptosis risk score (CRS) and other clinicopathological factors. **(A)** Results of ten-fold cross validation. **(B)** Box plots of risk score between two cuproptosis regulated patterns. **(C, D)** Univariable **(C)** and multivariable **(D)** regression results of CRS and other clinicopathological factors. **(E)** A nomogram was constructed by integrating CRS, age and tumor stage. **(F)** Calibration curves of our nomogram for 3 and 5 years. **(G–I)** The ROC curves of our nomogram for 1 **(G)**, 3 **(H)** and 5 **(I)** years respectively.

Supplementary Figure 3 Correlation between 14 novel genes and immune cell infiltration in TCGA-BLCA **(A)** and Xiangya cohort **(B)** respectively.

Supplementary Figure 4 Correlation between cuproptosis signature and immune cells infiltration in GSE32894 **(A)** and GSE13507 **(B)** respectively.

Supplementary Figure 5 Heatmaps of therapeutic targets from Drugbank database **(A)** and the enrichment scores of several therapeutic signatures **(B)** between different cuproptosis signature groups.

## Abbreviations

ICI, immune checkpoint inhibitor; BLCA, bladder carcinoma; TME, tumor microenvironment; CRS, cuproptosis risk score; TIL, tumor-infiltrating lymphocyte; MIBC, muscle invasive bladder carcinoma; NAC, neoadjuvant chemotherapy; RC, radical cystectomy; TMB, tumor mutational burden; CRT-1, copper transporter 1; DC, dendritic cell; BCG, bacillus Calmette-Guerin; FPKM, fragments per kilobase per million mapped fragments; TPM, transcripts per kilobase million; ICB, immune checkpoint blockade; ssGSEA, single sample gene set enrichment analysis; DEG, differentially expressed gene; FC, fold change; GO, gene ontology; KEGG, Kyoto Encyclopedia of Genes and Genomes; TIME, tumor immune microenvironment; TIP, tumor immunophenotype; LASSO, least absolute shrinkage and selector operation; scRNA-seq, single cell RNA sequencing; UMI, unique molecular identifier; PCA, principal component analysis; UMAP, uniform manifold approximation and projection; ROC, receiver operating characteristic; NSCLC, non-small cell lung cancer; NK, natural killer; Teff, T-effector; TIS, tumor inflammation signature; AUC, area under curve; Th1, type 1 helper; CR, complete response; PR, partial response; SD, stable disease; PD, progressive disease; EMT, epithelial-mesenchymal transition; LOX, lysyl oxidase; ECM, extracellular matrix; AOC3, amine oxidase copper containing 3; LUAD, lung adenocarcinoma; NMIBC, non-muscle invasive bladder cancer; FDA, Food and Drug Administration; ORR, objective response rate.
