# Supplementary figures and images for: Cuproptosis depicts tumor microenvironment phenotypes and predicts precision immunotherapy and prognosis in bladder carcinoma
Source: Front Immunol. 2022 Sep 23;13:964393. doi: 10.3389/fimmu.2022.964393 (PMC9540537; doi:10.3389/fimmu.2022.964393)

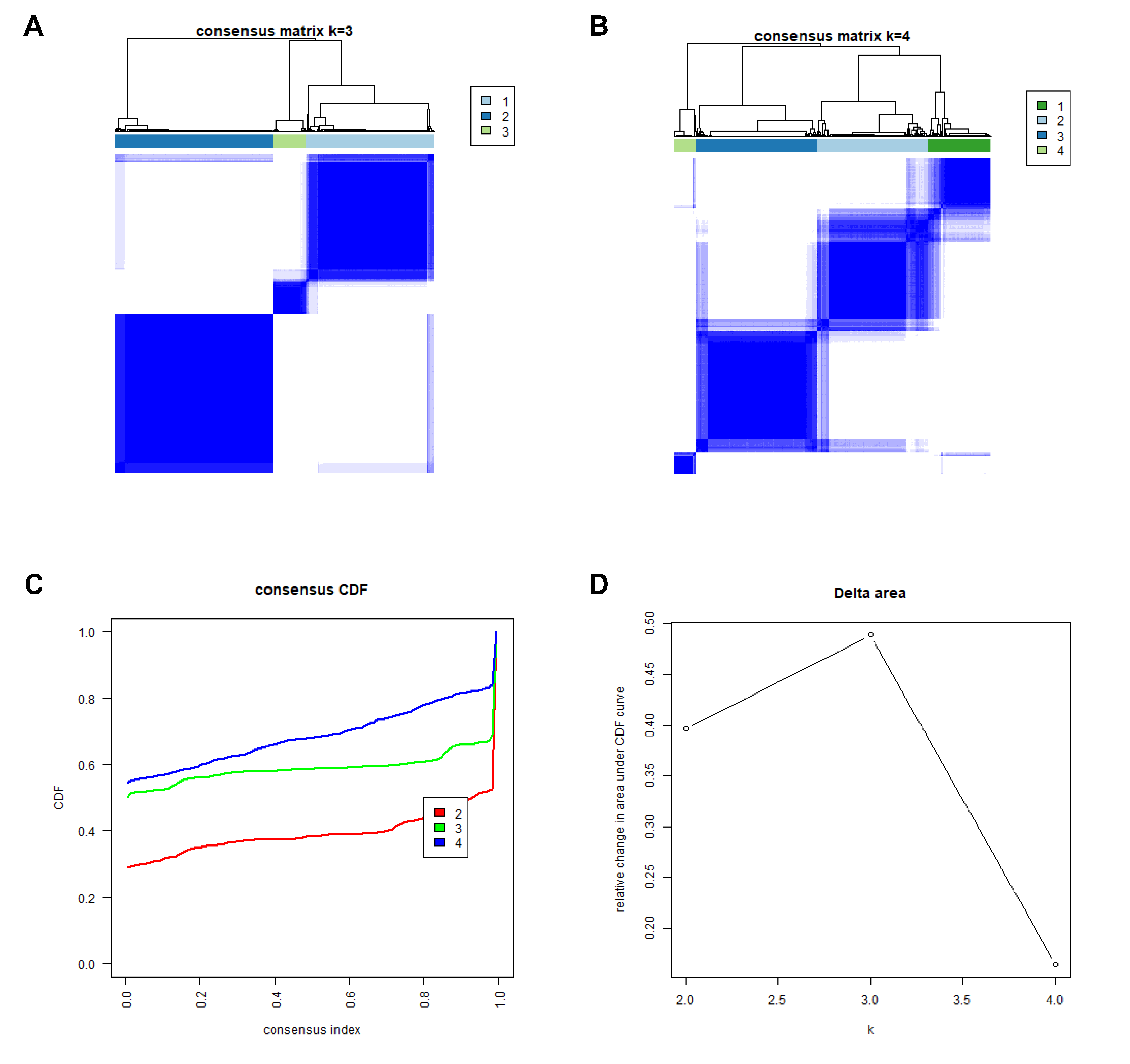

Supplement: Supplementary Figure 1 — Unsupervised clustering of 46 cuproptosis related genes in the TCGA-BLCA cohort. [file Image_1.tiff]

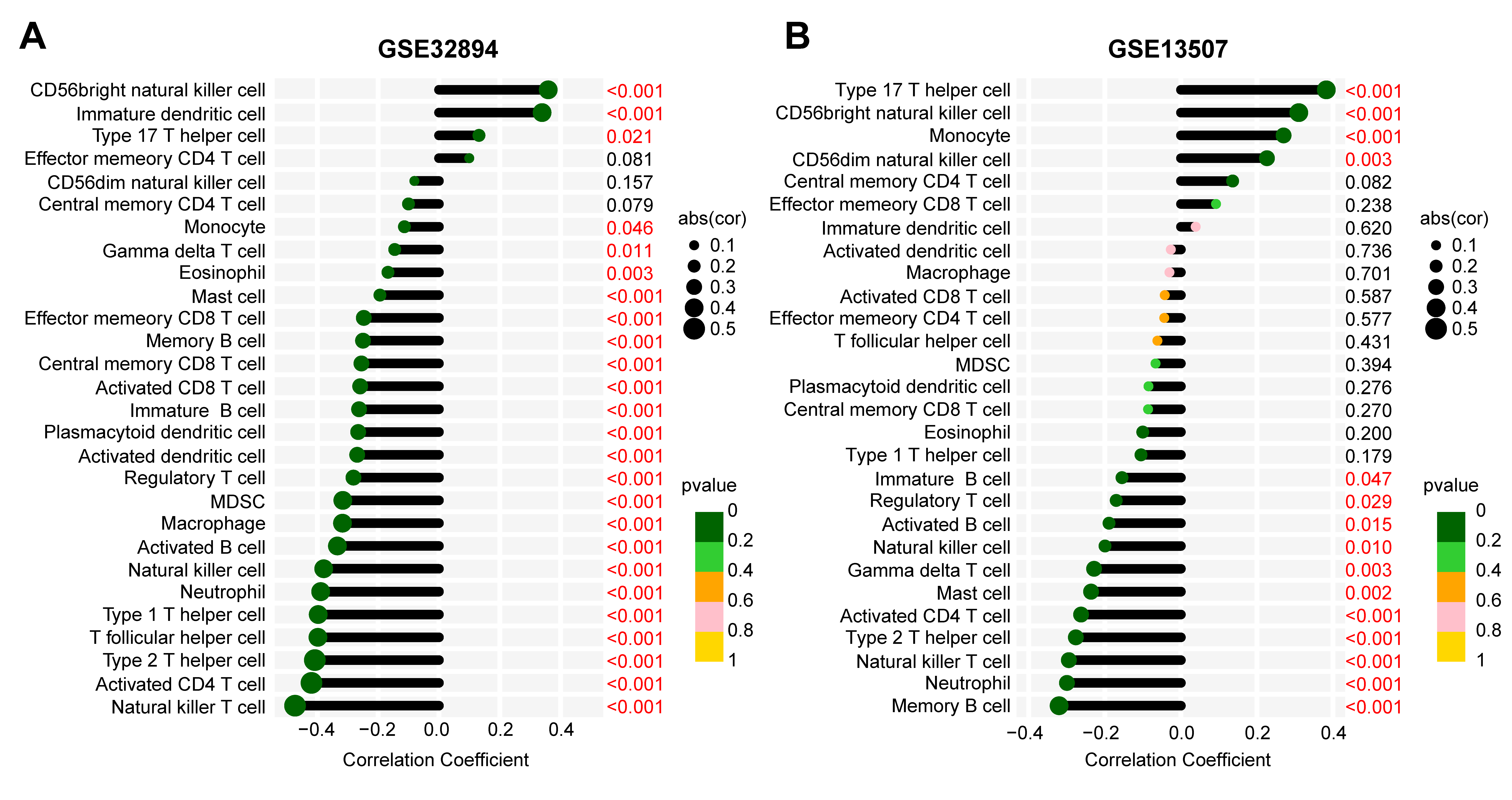

Supplement: Supplementary Figure 4 — Correlation between cuproptosis signature and immune cells infiltration in GSE32894 (A) and GSE13507 (B) respectively. [file Image_4.tiff]

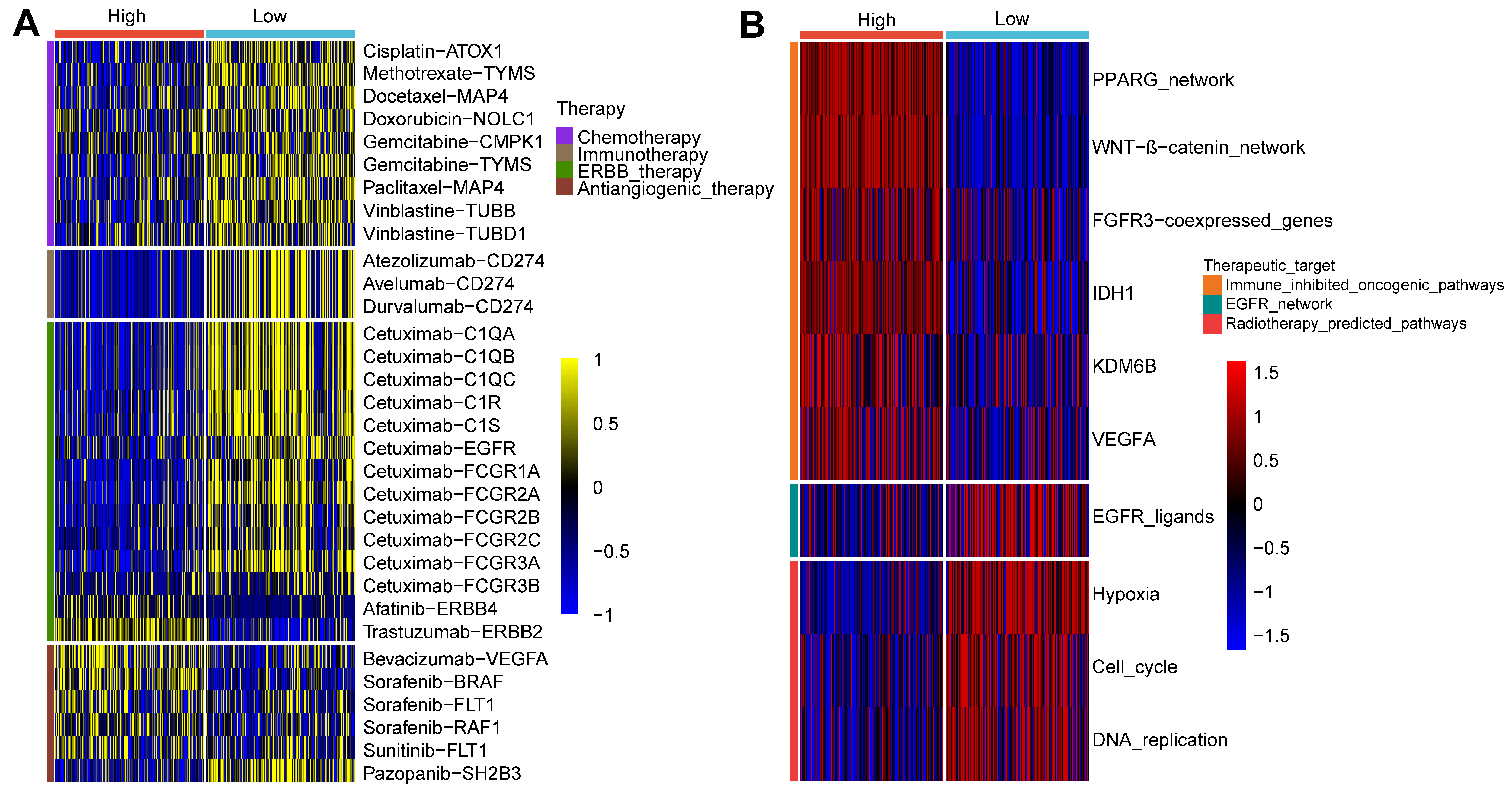

Supplement: Supplementary Figure 5 — Heatmaps of therapeutic targets from Drugbank database (A) and the enrichment scores of several therapeutic signatures (B) between different cuproptosis signature groups. [file Image_5.tiff]
